# Occupational Protein Contact Dermatitis: Two Case Reports

**DOI:** 10.1155/2010/489627

**Published:** 2010-08-30

**Authors:** Joana Rocha, Teresa Pereira, Artur Sousa-Basto, Celeste Brito

**Affiliations:** Department of Dermatology, Braga Hospital, Apartado 2242, 4701-965 Braga, Portugal

## Abstract

Protein contact dermatitis (PCD) is a contact dermatitis caused by high-molecular-weight proteins. This entity has been reported with increasing frequency, most commonly as occupational hand dermatitis in food handlers. Clinically, it is characterized by a chronic and recurrent dermatitis with erythema, scaling, and fissures with acute exacerbations occurring a few minutes after contact with offending allergen. We report two cases in confectioners who presented with chronic hand dermatitis.

## 1. Case Reports

**Case 1**

**Figure 1:**
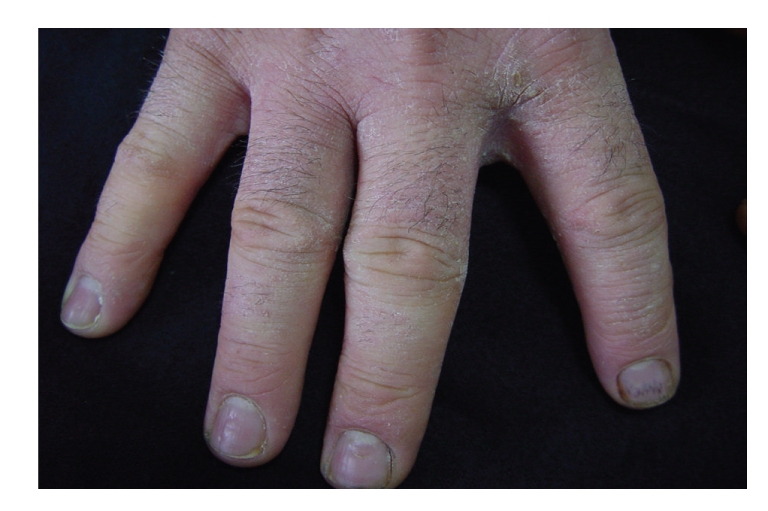
Hand eczema (Case 1).

A 27-year-old man, confectionery worker since he was 16 years old, presented with a history of chronic hand eczema, with acute exacerbations consisting of itching and erythema a few minutes after handling eggs. Some symptomatic relief was reported with glove protection. He had no respiratory, ocular, or digestive symptoms and no past history of atopy. His dermatitis improved during weekends and holidays but worsened when he returned to work. Clinical examination showed a highly pruriginous chronic eczema characterized by erythematous and scaly lesions and fissures in the back of the hands, fingers, and forearms, mainly on the right (Figure 1).

**Case 2**

**Figure 2:**
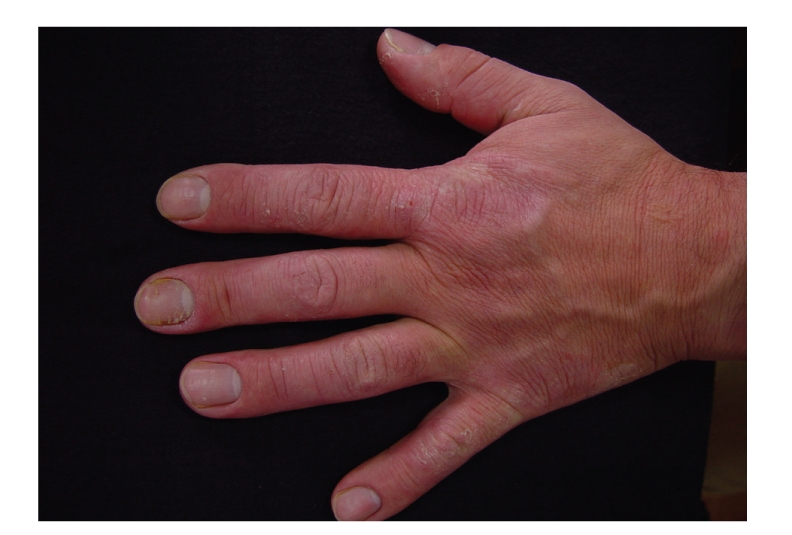
Hand eczema and paronychia (Case 2).

A 43-year-old confectionery worker (for 28 years) presented with chronic hand eczema. He referred to exacerbation of the symptoms a few minutes after handling eggs. He had no respiratory, ocular, or digestive symptoms and did not regularly wear protective gloves. He had family and personal history of atopic dermatitis. Clinical examination revealed erythematous and scaly highly pruriginous lesions and fissures in hands and forearms and paronychia (Figure 2).

## 2. Work-Up and Treatment

In both patients, patch test was performed with the Portuguese standard and bakery series and patients' own products. The allergens were applied to the upper back skin via an aluminium well and removed after 48 hours. There were no positive reactions after 72 hours.

Total serum IgE was high in the first case (316 UI/mL) and within normal range in the second.

RAST test was also performed (IMMULITE 2000 3gAllergy Specific IgE). Allergen-specific IgE was high for egg white, egg yolk, and wheat and rye flour in the first case (31,20 kU/L; 6,19 kU/L; 3,15 kU/L and 0,64 kU/L, resp.) and for egg white (2,26 kU/L) and egg yolk (0,391 kU/L) in the second. 

Skin prick tests were performed according to the international standards using the patients' own products. After 20 minutes, both patients developed a positive reaction to egg white and egg yolk. A positive reaction to rye flour was seen in the first case.

There was no worsening of the symptoms after skin tests. 

Based on the patch, prick, and RAST tests, PCD with immediate-type sensitization was diagnosed. 

Oral antihistamines and topical corticosteroids were prescribed with partial improvement of lesions, but both patients maintained acute exacerbations after contact with the responsible allergen. With regular use of protective gloves, the second patient's dermatitis' flares became rare.

## 3. Discussion

The term PCD was introduced in 1976 by Hjorth and Peterson [1] to describe a chronic and recurrent dermatitis caused by contact with proteinaceous material observed in sandwich makers. 

Its incidence is unknown, with an estimated prevalence varying from 5 to 10% [2]. 

It is characterized by a chronic and recurrent dermatitis with erythema, scaling, and fissures with immediate urticarial or vesiculous exacerbation occurring within few minutes after exposure to the causative protein allergen [3]. The hands are the most commonly affected site and usually in a diffuse manner. Some cases of chronic paronychia were considered a variety of PCD, with redness and swelling of the proximal nail folds [4]. 

It has been reported in many occupations (confectionery workers, bakers, cookers, kitchen workers, farmers, veterinarians, florists, and health workers) but mostly affects food handlers [4–9]. In 50% of the cases, an atopic history can be found [2, 10]. However, other conditions may also be responsible for the ability of high-molecular-weight substances to pass the epidermis and lead to sensitization [11]. Food can act both as irritant and allergen. Food handlers are at risk of developing dermatitis due to food sensitization or skin damage directly from their irritant effects, as well as from the wet environment that accompanies food preparation. When the allergen is volatile, respiratory and ocular symptoms (rhinitis, asthma, and conjunctivitis) can coexist [12, 13]. 

The pathogenesis of PCD is not entirely clear although most authors believe that it results from type I allergy to large-molecular-weight substances [10], that probably penetrate through a less effective skin barrier, either as a result of atopic eczema or irritant contact dermatitis. However, others believe that PCD results from combined type I (immediate) and type IV (delayed) IgE hypersensibility [14]. In our cases, as in the original cases of Hjorth and Roed-Peterson, PCD may have been caused by an IgE-mediated immediate allergy combined with irritant factors. 

The clinical picture, positive work relation, and allergological tests lead to the diagnosis of PCD, but standardized diagnostic criteria are not yet available. Diagnosis requires skin tests (preferably performed with fresh material), particularly open tests, prick tests, or scratch tests [2]. Positive reactions are observed after a few minutes. In some cases, specific IgE to the substance can be detected. However, RAST tests are not as sensitive as skin tests and are not available for every suspected allergen [15]. Although PCD presents clinically as chronic eczema, patch tests are usually negative [2].

Both of our patients had the clinical picture of egg yolk and egg white PCD, associated with positive prick and RAST tests. Although the first patient had positive RAST test to wheat and rye flour and a positive prick test to rye flour, he did not report acute exacerbations after handling flour alone, and he denied respiratory and ocular symptoms. 

In conclusion, the presence of PCD in food handlers with chronic eczema is not infrequent. In every case of occupation-related contact dermatitis, a careful allergologic evaluation of type I allergies should be performed. In PCD suspects, particularly food handlers with hand eczema, it is important to perform a prick test with their own food, especially in the presence of a history of immediate irritation after contact with certain foods. The detection of the causative allergen and its avoidance may lead to the resolution of the dermatitis.
